# Identification of 13 novel *USH2A* mutations in Chinese retinitis pigmentosa and Usher syndrome patients by targeted next-generation sequencing

**DOI:** 10.1042/BSR20193536

**Published:** 2020-01-21

**Authors:** Ling-hui Qu, Xin Jin, Yan-ling Long, Jia-yun Ren, Chuang-huang Weng, Hai-wei Xu, Yong Liu, Xiao-hong Meng, Shi-ying Li, Zheng-qin Yin

**Affiliations:** 1 Department of Ophthalmology, The 74th Army Group Hospital, Guangzhou 510318, China; 2 Department of Ophthalmology, General Hospital of Chinese People’s Liberation Army, Beijing 100853, China; 3 Southwest Hospital/Southwest Eye Hospital, Third Military Medical University, (Army Medical University), Chongqing 400038, China

**Keywords:** Non-syndromic retinitis pigmentosa, USH2A gene, Usher syndrome

## Abstract

**Background**: The *USH2A* gene encodes usherin, a basement membrane protein that is involved in the development and homeostasis of the inner ear and retina. Mutations in *USH2A* are linked to Usher syndrome type II (USH II) and non-syndromic retinitis pigmentosa (RP). Molecular diagnosis can provide insight into the pathogenesis of these diseases, facilitate clinical diagnosis, and identify individuals who can most benefit from gene or cell replacement therapy. Here, we report 21 pathogenic mutations in the *USH2A* gene identified in 11 Chinese families by using the targeted next-generation sequencing (NGS) technology.

**Methods:** In all, 11 unrelated Chinese families were enrolled, and NGS was performed to identify mutations in the *USH2A* gene. Variant analysis, Sanger validation, and segregation tests were utilized to validate the disease-causing mutations in these families.

**Results:** We identified 21 pathogenic mutations, of which 13, including 5 associated with non-syndromic RP and 8 with USH II, have not been previously reported. The novel variants segregated with disease phenotype in the affected families and were absent from the control subjects. In general, visual impairment and retinopathy were consistent between the USH II and non-syndromic RP patients with *USH2A* mutations.

**Conclusions:** These findings provide a basis for investigating genotype–phenotype relationships in Chinese USH II and RP patients and for clarifying the pathophysiology and molecular mechanisms of the diseases associated with *USH2A* mutations.

## Introduction

Retinitis pigmentosa (RP; OMIM 226800) is one of the most common incurable ocular diseases and can lead to severe visual impairment. It is characterized by clinical and genetic heterogeneity. Patients initially experience night blindness and visual field constriction resulting from rod degeneration, followed by deterioration of central vision owing to the loss of cone function. The global prevalence of RP is 1:3000–1:7000 [1]. Most patients have non-syndromic RP, while others suffer from associated syndromes. The most common of these is Usher syndrome (USH) [2], an autosomal recessive disorder that has a prevalence of 3.2–6.2/100000 and is characterized by sensorineural hearing loss (HL), visual impairment due to RP, and variable vestibular dysfunction with heterogeneous clinical and genetic manifestations [3,4]. Mutations in the Usher syndrome 2A (*USH2A*) gene give rise to two distinct phenotypes, USH type II (USH II) and non-syndromic RP, which account for 55–90% [2] and 12–25% [5] of cases, respectively.

The USH2A protein sequence contains laminin epidermal growth factor and fibronectin type III motifs, which are also observed in the basal lamina, extracellular matrix (ECM), and cell adhesion proteins. In the eye, both the Bruch’s membrane and interphotoreceptor cell matrix are enriched in ECM proteins, which play an important role in cochlear hair cell development and photoreceptor maintenance [6].

With advances in the fields of genetic engineering and stem cell research, gene therapy and photoreceptor transplantation have emerged as promising treatment strategies for patients with *USH2A* mutations [7–9]. Identifying individuals who will most benefit from these therapies is critical for successful treatment outcomes; this can be accomplished by examining the association between genetic defects and clinical phenotypes. Targeted-exome sequencing (TES) based on next-generation sequencing (NGS) is a powerful, robust, precise, and cost-effective method for detecting genetic mutations in large genomic regions. However, only a small number of studies have been published on the *USH2A* mutation spectrum in Chinese RP and USH patients [10–17]. The genetic characteristics of Chinese USH patients differ from those of the other populations. For example, the mutation c.8559- 2A > G in *USH2A* accounts for 19.1% in a Chinese USH II cohort [10], and 26% of all Western Japanese USH patients, but was never observed in Europeans. Furthermore, the full extent of genotype–phenotype correlations between *USH2A* mutations and RP and USH II symptoms in the Chinese population has not been elucidated.

In the present study, we used NGS-based TES to investigate mutations in the *USH2A* gene and identified 13 novel mutations in 5 USH and 6 RP families from China. The variants segregated with disease phenotype in the affected families and were not detected in the healthy controls. We also examined the genotype–phenotype correlations between these novel *USH2A* mutations and observed clinical symptoms.

## Materials and methods

### Pedigrees and controls

**Figure 1:**
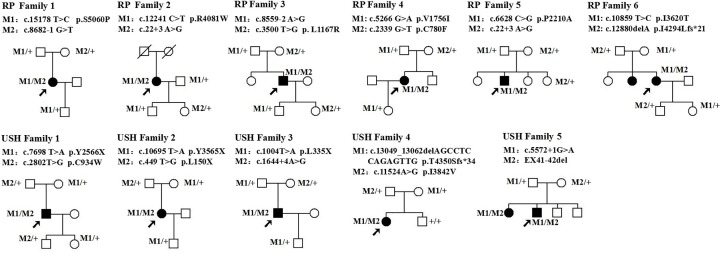
Pedigrees of families with non-syndromic RP or USH II Squares and circles indicate men and women, respectively, and dark symbols represent the affected individuals. The probands are indicated by an arrow. +, wild-type; M, mutation.

The study was conducted in accordance with the guidelines of the Declaration of Helsinki, and the protocol was approved by the Ethics Committee of Southwest Hospital, Third Military Medical University (Army Medical University), Chongqing, China. Written informed consent was obtained from all participants. Detailed family history was obtained through interviews with patients and their relatives (Figure 1). Ophthalmic examination included the best-corrected visual acuity assessment, dilated fundus examination, colour fundus photography, fundus autofluorescence (FAF) imaging, optical coherence tomography (OCT), and full-field electroretinogram (ERG). The ERG was performed according to the standards of the International Society for Clinical Electrophysiology. Abnormal cone function was assessed by light-adapted 30-Hz 3.0 ERG, and rod ERG abnormality was assessed by dark-adapted 0.01-Hz 10.0 ERG [18].

Audiologic assessment was also performed for each patient. The severity of HL was classified as mild (20–40 dB), moderate (41–70 dB), severe (71–90 dB), or profound (>91 dB) [19]. Vestibular function was evaluated based on the medical history related to childhood motor development [20] and results of caloric and rotary chair tests [21]. Five families showed congenital hearing impairment based on audiologic or vestibular function test results and were diagnosed with USH II. Six families did not have childhood-onset hearing impairment and were classified as non-syndromic RP families.

A group of 200 matched control subjects was also recruited for the study; these individuals were free of any symptoms of HL, visual impairment, or any personal or family history of known inherited disease. All 11 pedigrees and control subjects in the present study were from Southwestern China. Peripheral blood samples were collected from the affected patients, their unaffected kin, and healthy control subjects.

### DNA target capture and NGS

A capture panel of retinal disease (RD) genes including 103 known RD genes was developed by our group [14,22–23]. A sequence capture array (Roche NimbleGen, Madison, WI, U.S.A.) from the Beijing Genomics Institute, ShenZhen, was used to capture the coding exons of 103 RD genes associated with hereditary blindness or for whole-exome sequencing to identify RD-related mutations.

### Filtering of detected variants

To detect the potential pathogenic variants of *USH2A* in the probands, we applied the filtering criteria to generate clean reads (with lengths of 90 bp) for further analysis and then aligned them with the human reference genome in the National Center for Biotechnology Information (NCBI) database (v.hg19; http://www.ncbi.nlm.nih.gov/projects/genome/assembly/grc/) using the Burrows Wheeler Aligner Multi-Vision software package. The single-nucleotide variants and insertions/deletions (indels) were identified using the software programs SOAPsnp and Samtools Indel Genotyper, respectively, based on the data obtained from the NCBI Database of Single Nucleotide Polymorphisms (http://hgdownload-test.cse.ucsc.edu/goldenPath/hg19/database/), the HapMap project (ftp://ftp.ncbi.nlm.nih.gov/hapmap), and the 1000 Genome Project (ftp://ftp.1000genomes.ebi.ac.uk/vol1/ftp).

### PCR and Sanger sequencing

All novel mutations were validated by PCR and Sanger sequencing. DNA was obtained from the peripheral blood of family members (Figure 1) using the QIAamp DNA Blood Midi Kit (Qiagen, Hilden, Germany). Thereafter, a familial segregation analysis was performed by PCR and Sanger sequencing. The 200 healthy matched control subjects were also screened for the novel variants by Sanger sequencing.

### Pathogenicity assessment of novel mutations

Null variants were defined as those that were predicted to introduce a premature termination codon or affect pre-mRNA splicing or as frameshift mutations that have been classified as pathogenic. Novel missense variants were described as pathogenic based on their frequency in controls (<0.236%), segregation with disease, bioinformatics analysis of their pathogenicity (using the PolyPhen2 and SIFT tools available online), and conservation [21,24].

## Results

### Clinical characteristics of patients

The study population comprised 11 unrelated Chinese families—six with non-syndromic RP and five with USH II—residing in Southwestern China. All 11 families showed a genotype pattern suggesting a recessive mode of inheritance. The five probands with USH II had a mean age of 42.6 years (range: 23–60 years). The mean onset age was 12.4 years (range: 7–20 years). The USH II probands F2-II-1, F3-II-1, and F4-II-1 had initial symptoms of almost simultaneous night blindness and poor hearing; F2-II-1 had experienced night blindness from the age of 12 years; and F5-II-1 had poor hearing from the age of 20 years (Table 1). The mean age of the six RP probands at the time of diagnosis was 39.8 years (range: 33–57 years), and the mean onset age from the time of night blindness was 21.7 years (range: 7–39 years) (Table 1). The USH II patients tended to be younger than the RP patients at the time of symptom onset (i.e. night blindness and poor hearing), although the two groups were diagnosed at a similar age.

**Table 1:** Clinical manifestations of the proband patients

Proband	USH F1-II-1	USH F2-II-1	USH F3-II-1	USH F4-II-1	USH F5-II-2	RP F1-II-1	RP F2-II-1	RP F3-II-2	RP F4-II-1	RP F5-II-2	RP F6-II-3
Consult age (Y); gender	47; M	33; F	50; M	23; F	60; M	33; F	57; F	36; M	34; F	40; M	39; F
Onset age (Y); presenting symptom	12; night blindness	13; night blindness, poor hearing	10; night blindness, poor hearing	7; night blindness, poor hearing	20; poor hearing	28; night blindness	16; night blindness	11; night blindness	7; poor vision	39; night blindness	29; night blindness
Onset age for NB (Y)	12;	13;	10;	7;	45;	28	16	11	7	39	29
Deaf	Moderate	Severe	Severe	Severe	Severe	NO	NO	NO	NO	NO	NO
Vestibular defect	NO	NO	NO	NO	NO	NO	NO	NO	NO	NO	NO
BCVA (R/L)	0.03/ 0.15	0.6/0.7	0.4/0.6	0.4/0.4	0.7/0.5	0.8/ 0.8	0.7/0.5	0.4/0.3	0.1/HM	0.15/0.5	0.7/0.8
FERG findings	Extinguished rod and cone	Extinguished rod and cone	Extinguished rod and cone	Extinguished rod and cone	Extinguished rod and cone	Severely reduced rod and cone	Extinguished rod and cone	Extinguished rod and cone	Extinguished rod and cone	Extinguished rod and cone	Severely reduced rod and cone
OCT	Almost absent photoreceptor inner segment ellipsoid band	Preserved ellipsoid zone at central macula fovea	Preserved ellipsoid zone at central macula fovea	Preserved ellipsoid zone at central macula fovea	Preserved ellipsoid zone at central macula fovea	Preserved ellipsoid zone at macula	Preserved ellipsoid zone at central macula fovea	Absent outer retina layers with thinning of the retina	Absent outer retina layers with severely thinning of the retina	Absent outer retina layers with severely thinning of the retina	Preserved ellipsoid zone at central macula fovea
Clinical diagnosis	USH2	USH2	USH2	USH2	USH2	RP	RP	RP	RP	RP	RP
Comments	NO	Wearing hearing aids	NO	Wearing hearing aids	NO	NO	NO	NO	L:macular coloboma	NO	NO

Abbreviations: BCVA, best corrected visual acuity; F, female; FERG, full-field ERG; L, left eye; M, male; NB, night blindness; R, right eye, Y, years.

**Figure 2:**
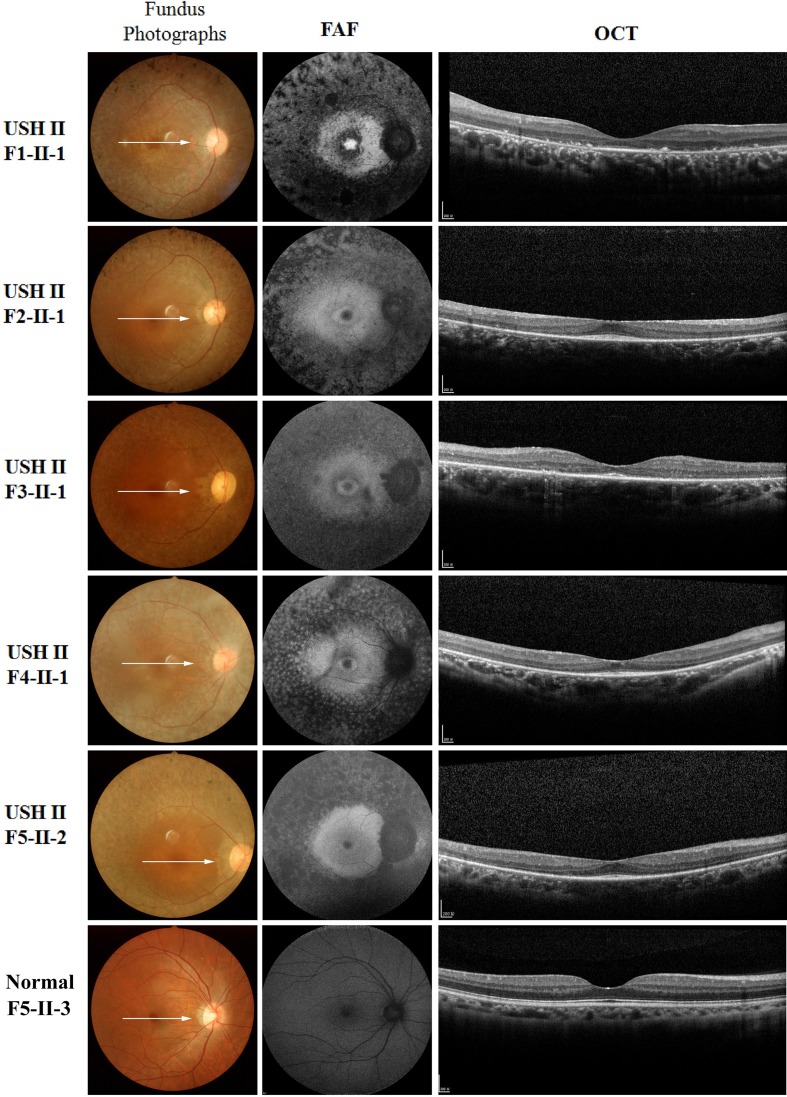
Fundus photography, FAF imaging, and foveal OCT scans of probands from the USH II families Attenuation of retinal arterioles, atrophy of retinal pigment epithelia, and waxy pale discs can be seen in all fundus photographs. USH II F1-II-1: Central hyperautofluorescence can be seen by FAF imaging, whereas the photoreceptor inner segment ellipsoid band is absent from the OCT scan. USH II F2-II-1, F3-II-1, F4-II-1, and F5-II-2: A hyperautofluorescent ring can be seen by FAF imaging, and a preserved photoreceptor inner segment ellipsoid band in the area was detected within the hyperautofluorescent ring in the OCT scan. USH II F5-II-3: The non-affected family member showed normal fundus and FAF; the OCT scan showed an intact photoreceptor inner segment ellipsoid band and regular structure in the macular. The white arrow indicates the OCT scanning direction.

**Figure 3:**
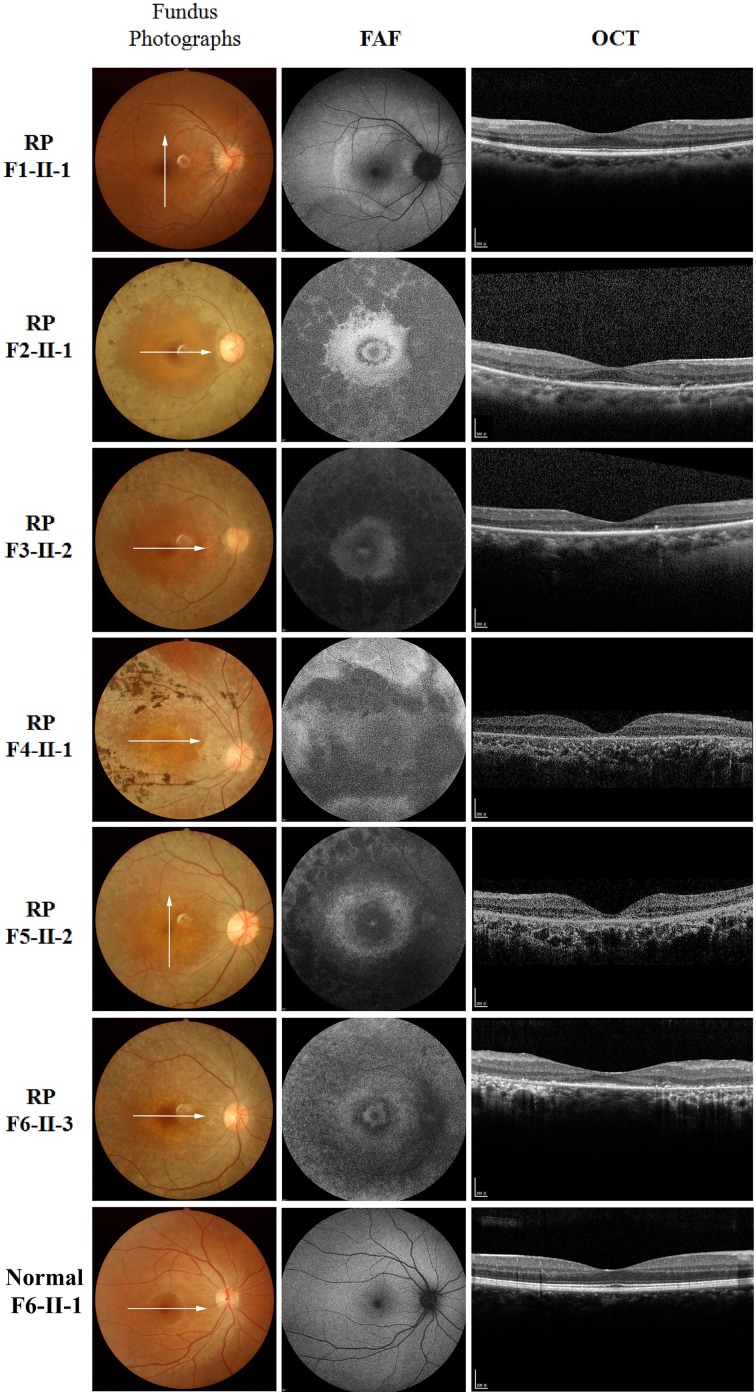
Fundus photography, FAF imaging, and foveal OCT scans of probands from non-syndromic RP families The fundus photograph shows the typical retinopathy of RP. RP F1-II-1: FAF imaging revealed completely preserved autofluorescence except for slightly higher autofluorescence surrounding the macula; a complete photoreceptor inner segment ellipsoid band was detected in the OCT scan. RP F2-II-1 and F6-II-3: A hyperautofluorescent ring and preserved photoreceptor inner segment ellipsoid band in the fovea can be seen by FAF imaging and OCT scanning. RP F3-II-2, F4-II-1, and F5-II-2: Severely decreased autofluorescence and absent outer retinal layers with thinning of the retinal pigment epithelium/Bruch’s membrane complex band were observed by FAF imaging and OCT scanning. RP F6-II-1: The non-affected family member showed normal fundus and FAF; the OCT scan showed an intact photoreceptor inner segment ellipsoid band and regular structure in the macular. The white arrow indicates the OCT scanning direction.

All probands showed typical RP features in the fundi, including pigmentary changes in the peripheral and mid-peripheral retina, attenuated arteriolar vessels, and pallor of the optic disc (Figures 2 and 3). All patients, except for the RP F1 (F1-II-1) and RP F6 (F1-II-3) probands, had no rod or cone responses in the ERG (Table 1). In the audiologic examination, a pure tone audiogram revealed bilateral downward-sloping moderate-to-severe HL in all five USH II probands (Table 1). The *USH2A*-associated USH II and non-syndromic RP patients showed similar retinopathy. Consistent with a previous report [25], four USH II (USH II F2-II-1, F3-II-1, F4-II-1, and F5-II-2) and two RP (RP F2-II-1 and F6-II-3) probands showed preserved central macular autofluorescence surrounded by a high-density ring of variable diameter (hyperautofluorescent ring) in FAF images (Figures 2 and 3). The USH II F1 proband (F1-II-1) had an abnormally high signal in the fovea (central hyperautofluorescence). Three RP probands (RP F3-II-2, F4-II-1, and F5-II-2) had widespread, severely diminished autofluorescence corresponding to retinal pigment epithelial atrophy. The RP F1 proband (F1-II-1) had the most fully preserved autofluorescence, with a slightly higher signal surrounding the macula.

An overlay of FAF and OCT images (Figures 2 and 3) revealed that the hyperautofluorescent ring represented a boundary between the relatively preserved photoreceptor inner segment ellipsoid band and diseased retinal tissue. A high degree of correlation was observed between the lateral extent of the preserved ellipsoid band over the central macula and ring diameter or width along the same OCT scanning plane. The central hyperautofluorescence (USH F1-II-1) showed a near-absent photoreceptor inner segment ellipsoid band by OCT, and the severely decreased autofluorescence (RP F3-II-2, F4-II-1, and F5-II-2) reflected an absence of the outer retinal layers with thinning of the retinal pigment epithelium/Bruch’s membrane complex band.

### Identification of *USH2A* gene mutations

**Figure 4:**
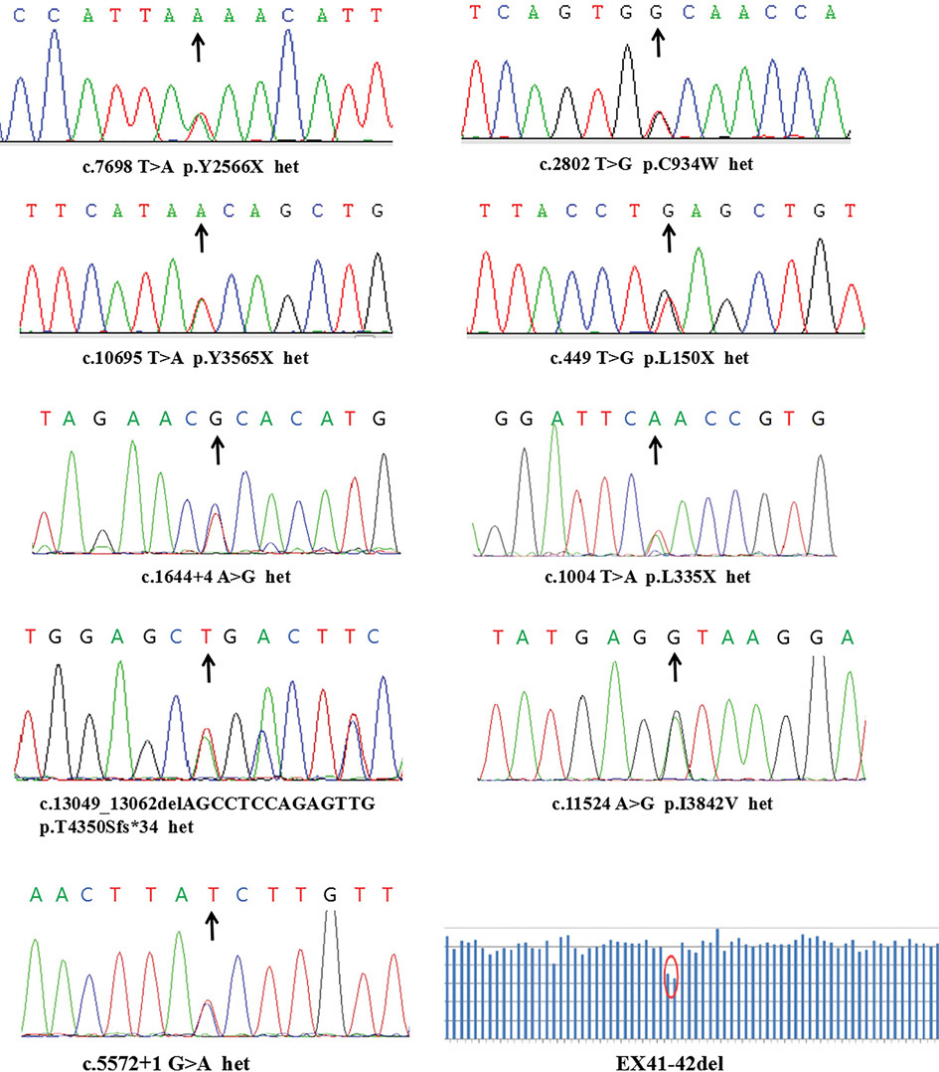
*USH2A* mutations associated with USH II confirmed by Sanger sequencing The deletion of exons 41 and 42 of the *USH2A* gene was detected (last image, right, bottom row). Arrows indicate mutations in the pathogenic genes. Abbreviation: het, heterozygous.

**Figure 5:**
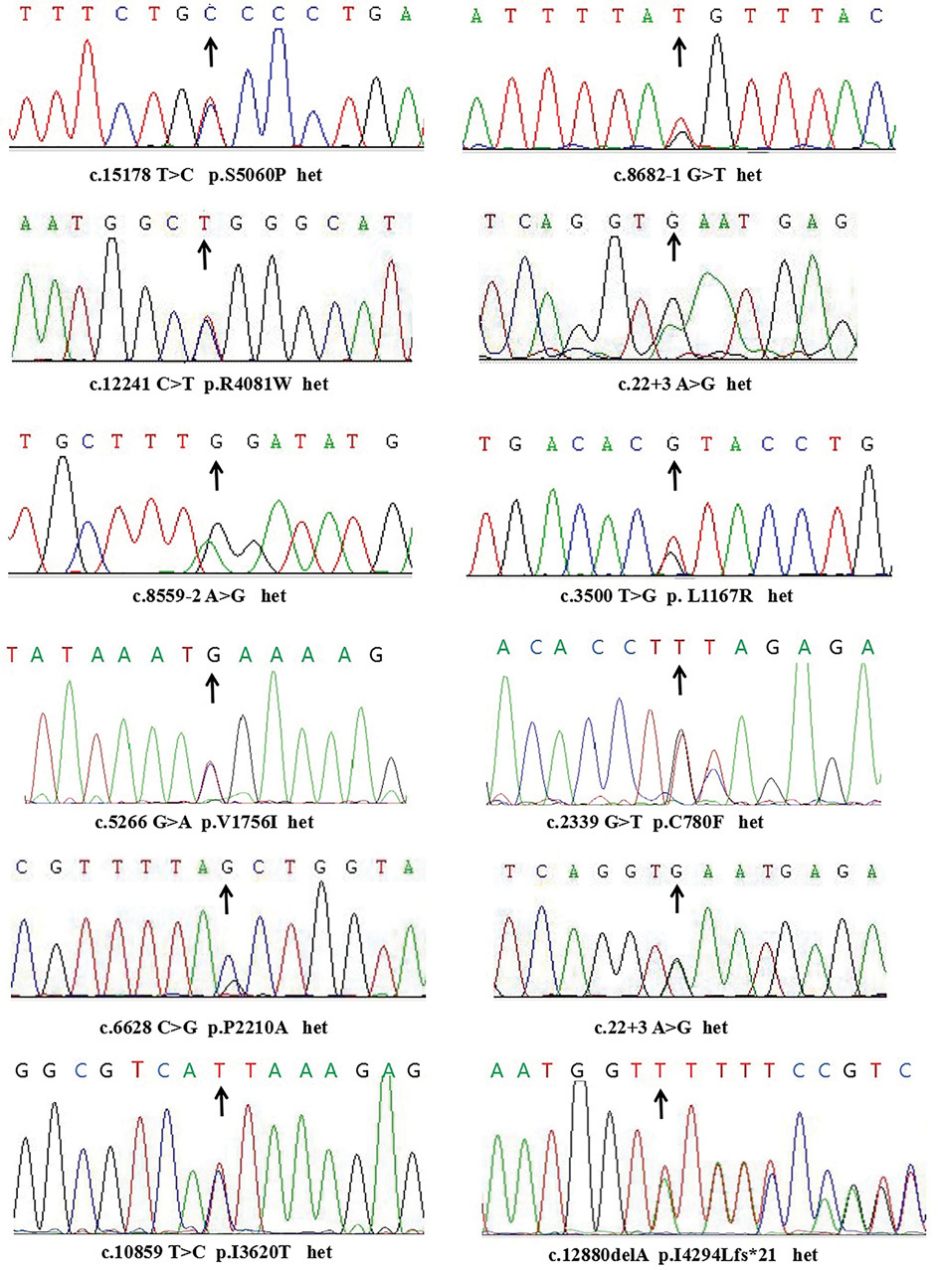
*USH2A* mutations associated with non-syndromic RP confirmed by Sanger sequencing Arrows indicate mutations in the pathogenic genes. Abbreviation: het, heterozygous.

The targeted NGS data for the 11 patients with non-syndromic RP and USH II were analyzed based on our genetic variation pathogenicity assessment [14,22–23]. After variant calling and data filtering, we identified 10 compound mutations in five USH II patients and 11 compound mutations in six RP patients; the new splice-site mutation c.22+3 A>G was detected twice in RP F2 and RP F5 (Table 2). These compound mutations included non-synonymous substitution, splice-site, point-nonsense mutation, frameshift deletion, and large-fragment deletion. In all, we identified 21 *USH2A* mutations, of which 13 have not been previously reported. The variants were confirmed by Sanger sequencing (Figures 4 and 5).

**Table 2:** *USH2A* mutations identified by NGS analyses

Family	Proband	Phenotype	Gene	Exon or Intron	Nucleotide change	Amino acid change	Mutation type^1^	SIFT/Polyphen2^2^	Reported or Novel
USH-F1	II -1	USH II	*USH2A*	EX41	c.7698 T>A	p.Y2566X	Nonsense; het	N.P	Novel
			*USH2A*	EX13	c.2802 T>G	p.C934W	Missense; het	N.P	Reported
USH-F2	II -1	USH II	*USH2A*	EX54	c.10695 T>A	p.Y3565X	Nonsense; het	N.P	Novel
			*USH2A*	EX2	c.449 T>G	p.L150X	Nonsense; het	N.P	Novel
USH-F3	II -1	USH II	*USH2A*	EX6	c.1004 T>A	p.L335X	Nonsense; het	N.P	Novel
			*USH2A*	EX9	c.1644+4A>G	_	Splicing; het	N.P	Novel
USH-F4	II -1	USH II	*USH2A*	EX63	c.13049_13062delAGCCTC CAGAGTTG	p.T4350Sfs*34	Deletion; het	N.P	Novel
			*USH2A*	EX59	c.11524 A>G	p.I3842V	Missense; het	0.263/0.015	Novel
USH-F5	II -2	USH II	*USH2A*	EX28	c.5572+1G>A	_	Splicing; het	N.P	Reported
			*USH2A*	EX41-42	EX41-42del	_	Deletion; het	N.P	Novel
RP-F1	II -1	RP	*USH2A*	EX70	c.15178 T>C	p.S5060P	Missense; het	2.222/0.771	Reported
			*USH2A*	IN43	c.8682-1G>T	_	Splicing; het	N.P	Novel
RP-F2	II -1	RP	*USH2A*	EX62	c.12241 C>T	p.R4081W	Missense; het	4.484/0.720	Reported
			*USH2A*	IN22	c.22+3 A>G	_	Splicing; het	N.P	Novel
RP-F3	II -2	RP	*USH2A*	IN42	c.8559-2 A>G	_	Splicing; het	N.P	Reported
			*USH2A*	EX9	c.3500 T>G	p. L1167R	Missense; het	3.774/0.986	Novel
RP-F4	II -1	RP	*USH2A*	EX26	c.5266 G>A	p.V1756I	Missense; het	0.079/0.005	Reported
			*USH2A*	EX13	c.2339 G>T	p.C780F	Missense; het	10.752/0.999	Novel
RP-F5	II -2	RP	*USH2A*	EX34	c.6628 C>G	p.P2210A	Missense; het	5.236/0.1	Reported
			*USH2A*	IN22	c.22+3 A>G	_	Splicing; het	N.P	Novel
RP-F6	II -3	RP	*USH2A*	EX55	c.10859 T>C	p.I3620T	Missense; het	3.774/0.977	Reported
			*USH2A*	EX63	c.12880delA	p.I4294Lfs*21	Deletion; het	N.P	Novel

1 Mutation type: het, heterozygous; hom, homozygous.

2 SIFT/Polyphen2: N.P (not predicted).

In the five USH II families, ten different *USH2A* compound mutations were recorded, of which eight mutations, including four nonsense, one splice-site, one missense, one deletion, and one large-fragment deletion, were newly identified (Table 2). All USH II patients harbored two truncating mutations or one non-truncating and one truncating mutation.

Two variants that might be associated with the disease were present in USH II F1; these included the novel nonsense mutation c.7698 T>A in exon 41 and the previously reported c.2802 T>G missense mutation in exon 13 [16]. The former mutation leads to a tyrosine substitution in codon 2566 (p.Y2566X), causing premature termination of *USH2A* mRNA translation. The USH II F2 family harbored the two novel nonsense mutations c.10695 T>A and c.449 T>G. The former was a tyrosine substitution at codon 3565 that introduced a premature termination codon (p.Y3565X), whereas the latter involved a leucine substitution at codon 150, a termination codon (p.L150X). In USH II F3, proband F3-II-1 was found to carry the novel compound heterozygous mutations c.1004 T>A and c.1644+4 A>G. The former led to the substitution of leucine with a termination codon at codon 335 (p.L335X), generating a truncated USH2A protein; the latter was a splice-site mutation that was predicted to create an ectopic splice site. In USH II F4, the novel heterozygous compound *USH2A* mutations c.13049_13062delAGCCTCCAGAGTTG and c.11524 A>G were identified in proband F4-II-1. The former mutation in exon 63 caused a threonine-to-serine substitution at codon 4350 that was expected to cause a frameshift and premature termination of translation after the insertion of 34 irrelevant amino acids starting from serine at position 4350 (p.T4350Sfs*34). The c.11524 A>G missense mutation in exon 59 led to the substitution of valine for isoleucine at codon 3842 (p.I3842V). In USH II F5, the compound heterozygous mutation c.5572+1 G>A and a large-fragment deletion in exons 41–42 (EX41–42del: 964 bp deletion) of *USH2A* were identified in proband F5-II-2; the former is a known splice-site mutation [26]. In the six non-syndromic RP families, we identified 12 different *USH2A* compound heterozygous mutations, of which six mutations, including three splice-site, two missense, and one deletion, were novel (Table 2). Among the six non-syndromic RP families, four (RP F1, F2, F3, and F5) had similar genotypes with one missense and one splice-site mutation. In RP F4, proband F4-II-1 had two missense mutations, whereas in RP F6, proband F6-II-3 carried one missense mutation and a truncation. The two new splice-site mutations c.8682−1 G>T and c.22+3 A>G were each predicted to introduce an ectopic splice site. The previously reported splice-site mutation c.8559-2 A>G was detected in proband F3-II-2 [10,14,20]. An online splice-site prediction software program indicated that this variant would abolish the splice acceptor site in exon 43, resulting in the skipping of this exon. The two new missense mutations c.3500 T>G and c.2339 G>T were predicted to be pathogenic. The c.3500 T>G mutation in exon 9 led to the substitution of leucine with arginine at codon 1167 (p. L1167R). The other mutation, c.2339 G>T in exon 13 led to the substitution of cysteine with phenylalanine at codon 780 (p.C780F). The new truncating mutation c.12880delA was predicted to result in a frameshift and premature termination of translation after a 21-amino acid insertion starting from leucine at codon 4294 (p.I4294Lfs*21).

### Analysis of mutation pathogenicity

Of the 21 identified mutations, 13 have not been previously described; of these, 10 mutations, including four nonsense and three splice-site mutations, two deletions, and one large-fragment deletion, were null variants (Table 2). The remaining three mutations were missense mutations that were predicted to be pathogenic based on the following evidence. First, they were not detected in the 200 healthy control subjects and were not in any of the public or our internal variant databases that together comprise the data from approximately 20000 individuals. Second, they are located within the functional domains of the *USH2A* gene. Third, an *in silico* analysis of the three variants predicted that they are detrimental mutations (Table 2). Finally, the variants co-segregated perfectly with the disease within the affected families according to a recessive pattern of inheritance (Figure 1), as determined by PCR and Sanger sequencing (Figures 4 and 5).

### Mutation spectrum in the USH2A protein

**Figure 6:**
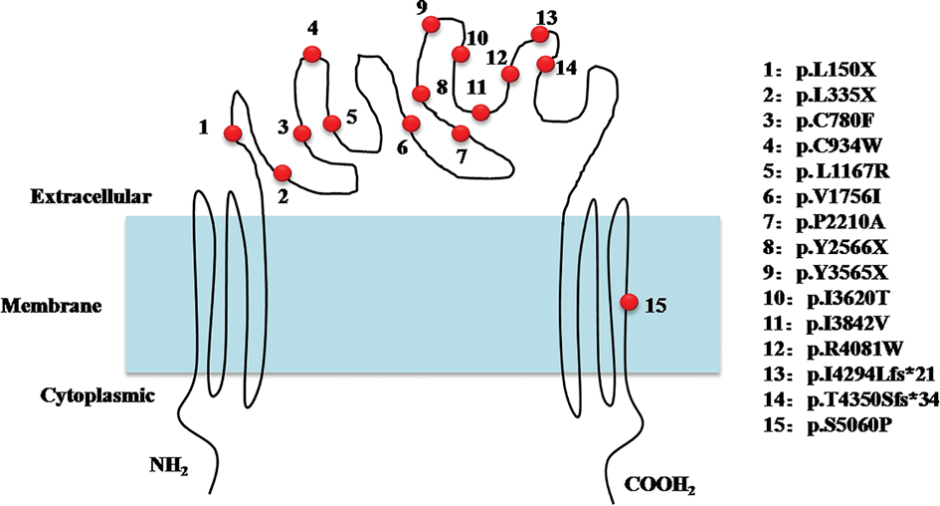
Predicted cellular distribution of the detected mutations *USH2A* gene encodes a transmembrane protein. Mutation p.S5060P is predicted in the membrane bound protein, other mutations are distributed in the exocytoplasmic region.

The distribution of these mutations did not significantly differ between the two kinds of RDs; the mutations were rather scattered over the whole sequence (Figure 6). Although the new splice-site mutation c.22+3 A>G was detected twice in our study, the most common reported mutation c.8559- 2A>G in Chinese USH II patients was also detected in our non-syndromic RP F3 family, and the mutations p.I4294Lfs*21 and p.T4350Sfs*34 were located in the same exon 63. We consider that the mutations identified in our study, including the new ones, do not suggest the presence of a mutation hotspot in the *USH2A* gene in the population examined. Nevertheless, larger clinical trials may help further unravel the genetic architecture of the Chinese population.

## Discussion

In the present study, we performed genetic diagnosis for USH II and non-syndromic RP cases arising from mutations in *USH2A* in 11 unrelated Chinese families by NGS and consequently identified 21 associated mutations, of which 13 were novel and definitively pathogenic. We also found that the USH II patients exhibited visual and hearing symptoms at an earlier age and had an earlier disease onset than the non-syndromic RP patients. Overall, our results demonstrate the clinical and genetic heterogeneity of USH II and non-syndromic RP are associated with *USH2A* mutations.

Molecular genetic testing can inform diagnosis, prognosis, and risk assessment for patients and their family members. *USH2A* mutations are present in most cases of USH II and non-syndromic RP. There are two isoforms of *USH2A*—a 170-kDa short isoform translated from 21 exons and a 580-kDa long isoform translated from an additional 51 exons. The former is the 5202-amino acid transmembrane usherin protein [27]. To date, approximately 1200 variants/mutations in the *USH2A* gene have been reported in patients with USH II or RP (see the *USH2A* mutations database: http://www.lovd.nl/USH2A). Owing to the large size of the *USH2A* gene, the Sanger sequencing of individual variants is costly and time-consuming. The targeted NGS technology enables massive coverage in the detection of DNA variants in inherited disorders—which is important for diagnostic purposes—with high efficiency at a relatively low cost.

In the present study, we identified 13 novel mutations in the *USH2A* gene. The six non-syndromic RP families were the carriers of one new mutation, with the others being previously known variants. In RP F1, the novel c.8682-1 G>T splice-site mutation was present in combination with the reported missense mutation c.15178 T>C. The c.15178 T>C, p. (Ser^5060^Pro) mutation was previously reported to be specific to non-syndromic RD along with c.2332 G>T, p. (Asp^778^Tyr); c.3724C>T, p. (Pro^1242^Ser); c.4378 G>A, p. (Gly^1460^Arg); c.8546 G>T, p. (Gly^2849^Val); c.6904_6920dup17, p. (Gln^2307^Hisfs*25); and c.12580 T>C, p. (Cys^4194^Arg) [25]. Indeed, in our study, the proband showed only retinal symptoms, although additional clinical and experimental data are needed to confirm the RD-specific nature of these alleles. The c.10859 T>C missense mutation in non-syndromic RP F6 has also been detected in a Japanese non-syndromic RP patient. In our study, the patients with this mutation showed similar characteristics. In RP F3, the splice-site mutation c.8559-2A>G has been described in Japanese [19,20] and Chinese [10,14] USH II patients; however, this mutation is a frequent founder variant in Japanese USH II patients [20] and the most common mutation in Chinese USH II patients [10]. We have previously reported the presence of this mutation in combination with c.9570+1 G>A, another splice-site variant, in a USH II family [14]. However, in the present study, this mutation was detected in a non-syndromic RP F3 family. Ten different *USH2A* compound mutations were recorded in the five USH II families, of which eight were newly identified. The c.2802 T>G mutation (p.C934W) is the second common *USH2A* mutation in Chinese USH II patients [10]. It has also been observed in a Chinese family in which the proband and his son showed typical USH II features, while the proband’s daughter suffered from RP without deafness or vestibular dysfunction, despite having the same genotype as the affected son [16]. These findings suggest that different mutations in a gene can give rise to distinct phenotypes and that the same gene mutation can also result in variable phenotypes. Perhaps the polygenetic/modifier genes, such as gene *PDZD7* or other undetected modifier genes, play a role in the disease phenotypes. Those unknown modifier genes could be pivotal contributors specific to non-syndromic RD, or complete USH II [2]. The other reported mutation, c.5572+1G>A, has been detected in a Scandinavian USH II patient [26]. *USH2A* gene encodes a transmembrane protein, which has a two-membrane structure domain in both the N and C terminals (Figure 6). The defect in the usherin protein from congenital mutations may lead to the disorder of the connecting cilium in the photoreceptors and the hair bundle of auditory sensory cells, causing disease.

Few studies have been conducted on the visual prognosis and genotype–phenotype correlations in retinal degeneration arising from *USH2A* mutations. A Japanese study demonstrated that patients with *USH2A*-associated USH II showed an earlier decline in visual function and had a higher cumulative risk of visual impairment than those with non-syndromic RP [5]. Complete loss of the USH2A protein function predisposed subjects to USH II, but residual protein function could cause RP without HL. A Chinese study arrived at a similar conclusion; truncating mutations predicted to have severe functional consequences were more highly represented in USH II than in non-syndromic RP patients, who often carried partial protein-truncating mutations [13]. Another recent Chinese study reported that the USH II patients with two null mutations of *USH2A* had an early onset age of HL [10]. Additionally, the patients harbouring two null mutations of *USH2A* had more severe HL than the patients carrying two missense mutations. In contrast, no statistically significant difference was observed in the onset age of the visual defect among patients with different kinds of mutations. However, a study in the U.K. showed that the *USH2A*-associated USH II cases exhibited similar retinopathy to the *USH2A*-associated non-syndromic RP cases, although the audiological phenotype differed, and some *USH2A* mutations were predominantly associated with non-syndromic retinal degeneration (i.e. they were RD-specific) [25]. Consistent with the above reports, the USH II patients in our study had a younger age of onset of symptoms and similar retinopathy to the *USH2A*-associated non-syndromic RP patients. We also showed that FAF imaging is a clinically useful tool for assessing the status of retinal pigment epithelial cells, while OCT scanning shows the preserved photoreceptor inner segment ellipsoid band in the residual region within the normal FAF.

Our study has some limitations. First, the study population was relatively small. Second, we did not conduct a longitudinal study comparing visual impairment and retinopathy between the *USH2A*-associated USH II and non-syndromic RP patients, which was necessary for a more accurate clinical assessment and for elucidating genotype–phenotype correlations. Nonetheless, we identified 13 novel *USH2A* mutations in 11 Chinese USH II or non-syndromic RP families by gene panel-based NGS. Our findings expand the spectrum of known *USH2A* mutations and related clinical phenotypes in the Chinese population. We also demonstrated that the *USH2A*-associated USH II patients are younger at the time of symptom onset than the non-syndromic RP patients. Collectively, these findings provide a basis for a more accurate diagnosis of USH and non-syndromic RP and the application of gene therapy to diseases associated with *USH2A* mutations.

## Abbreviations

ECM: extracellular matrix
ERG: electroretinogram
FAF: fundus autofluorescence
HL: hearing loss
NGS: next-generation sequencing
OCT: optical coherence tomography
RD: retinal disease
RP: retinitis pigmentosa
TES: targeted-exome sequencing
USH: Usher syndrome
